# Diagnostic Value of Serum D-dimer, CA19-9, and CT Imaging Features in Pancreatic Ductal Adenocarcinoma and Benign Pancreatic Lesions

**DOI:** 10.7150/jca.111548

**Published:** 2025-06-12

**Authors:** Yuan Li, Yingying Cao, Yaping Zhang, Tao Zhou, Fan Xia, Shuai Ren, Zhongqiu Wang

**Affiliations:** Department of Radiology, Affiliated Hospital of Nanjing University of Chinese Medicine, Nanjing 210029, China.

**Keywords:** computed tomography, pancreatic ductal adenocarcinoma, D-dimer, carbohydrate antigen 19-9

## Abstract

**Background:** The distinction between pancreatic ductal adenocarcinoma (PDAC) and benign pancreatic lesions remains challenging. This study aimed to evaluate the utility of computed tomography (CT) imaging features and clinical characteristics in differentiating PDAC from benign pancreatic lesions.

**Methods:** In this retrospective study, a total of 97 patients with PDAC and 90 patients with benign pancreatic lesions were included. Various imaging features and clinical characteristics were assessed. Univariable and multivariable logistic regression analyses were conducted, and receiver operating characteristic (ROC) curves and their corresponding areas under the curve (AUCs) were assessed. The optimal cut-off value for D-dimer was determined using the Youden index. The DeLong test was employed to compare the AUCs of the ROC curves between different prediction models.

**Results:** The clinical and radiologic models achieved AUCs of 0.86 and 0.85, respectively. Moreover, the combined model demonstrated superior predictive performance compared to either model alone. This overall model included two significant clinical predictors (D-dimer and CA19-9) and three radiological predictors (lymph node enlargement, pancreatic atrophy, and cystic components). It yielded an AUC of 0.92 (95% CI: 0.88-0.95), with a sensitivity of 83.5% and specificity of 82.2%. In addition, the optimal cut-off value of D-dimer for differentiating PDAC from benign pancreatic lesions was found to be 0.84 mg/L.

**Conclusions:** The overall model including clinical and radiologic variables (e.g., serum D-dimer, CA19-9, lymph node enlargement, pancreatic atrophy, and cystic components) demonstrated higher sensitivity and specificity in differentiating PDAC from benign pancreatic lesions. Serum D-dimer may serve as a valuable adjunctive biomarker in the diagnosis of pancreatic cancer and may further enhance the diagnostic performance of CA19-9 when used in combination.

## Introduction

Pancreatic cancer remains one of the most lethal malignancies of the digestive system, with pancreatic ductal adenocarcinomas (PDAC) accounting for approximately 90% of all cases. The 5-year survival rate for pancreatic cancer is only 13%, making it the third-leading cause of cancer death in both men and women 1. Unfortunately, the incidence of pancreatic cancer continues to rise annually. A recent report predicts that, based on current trends, pancreatic cancer will become the second leading cause of cancer-related deaths by 2040 2. Although surgical resection remains the only potential curative treatment, most patients (> 80%) are diagnosed at an advanced stage, thus missing the opportunity for intervention 3. Due to the absence of specific symptoms, early and accurate diagnosis of pancreatic cancer is challenging, not only for improving prognosis but also for preventing unnecessary invasive procedures in patients with benign pancreatic lesions.

Imaging evaluation plays a crucial role in the initial decision-making process for patients with PDAC. Multidetector computed tomography (MDCT) is the primary imaging modality for suspected cases, providing excellent spatial and temporal resolution, along with widespread accessibility 4. The hallmark CT feature of PDAC is a hypoattenuating pancreatic mass. Secondary findings, including pancreatic duct dilatation, common bile duct dilatation, and pancreatic parenchymal changes, further support the diagnosis. When located in the pancreatic head, PDAC may obstruct both the pancreatic duct and the common bile duct, a phenomenon known as the “double duct” sign 5, 6. However, certain benign pancreatic lesions can exhibit imaging features like those of PDAC. Chronic mass-forming pancreatitis can present as a hypoattenuating, mildly enhancing mass in the pancreatic head, making it difficult to distinguish from PDAC, especially when calcifications are present. Additionally, pancreatic neuroendocrine tumors (pNETs) display variable imaging features and may mimic PDAC when appearing hypovascular or exhibiting rim enhancement 7. Moreover, PDAC with cystic degeneration and necrosis may exhibit imaging features that overlap with those of benign cystic-solid lesions, including intraductal papillary mucinous neoplasms (IPMNs), cystadenomas, and solid pseudopapillary tumors.

Serum carbohydrate antigen 19-9 (CA19-9) is a Lewis antigen belonging to the MUC1 protein family, expressed on the surface of cancer cells 8. Currently, CA19-9 is recognized as the most prominent serological biomarker for diagnosing PDAC. However, studies evaluating its use as an auxiliary diagnostic tool have demonstrated that CA19-9 lacks sufficient specificity and sensitivity for effective screening 9. CA19-9 is not exclusively expressed in PDAC; its level is also elevated in benign conditions, including liver cirrhosis, chronic pancreatitis, cholangitis, and bile duct obstruction 10. Moreover, external factors, such as jaundice and other inflammatory conditions, can affect the accuracy of CA19-9. To enhance its diagnostic performance, CA19-9 has been evaluated in combination with various other biomarkers in research studies 11, 12.

Since Trousseau et al first identified the association between cancer and thrombosis in 1865, the interplay between cancer and the coagulation pathway has been the focus of extensive investigation 13. Venous thrombosis has been recognized as the second leading cause of death in cancer patients, surpassed only by cancer progression 13. It has been established that PDAC has a distinct ability to induce a hypercoagulable state, often presenting as subclinical abnormalities in conventional coagulation tests 14, 15. D-dimer is a specific degradation product generated when crosslinked fibrin is degraded by plasmin, which plays a role in decomposing thrombus and maintaining vascular smoothness. It serves as a biomarker indicating the activation of coagulation and fibrinolysis. Elevated D-dimer levels have been reported in patients with pancreatic cancer 16. Its value as an independent prognostic marker for PDAC is well-established, although few studies have explored its diagnostic accuracy in PDAC 17.

In this study, we aimed to develop a predictive model that combines clinical and imaging features to provide a valuable tool for the early screening and adjunctive diagnosis of PDAC.

## Materials and Methods

### Study patients

**Figure 1:**
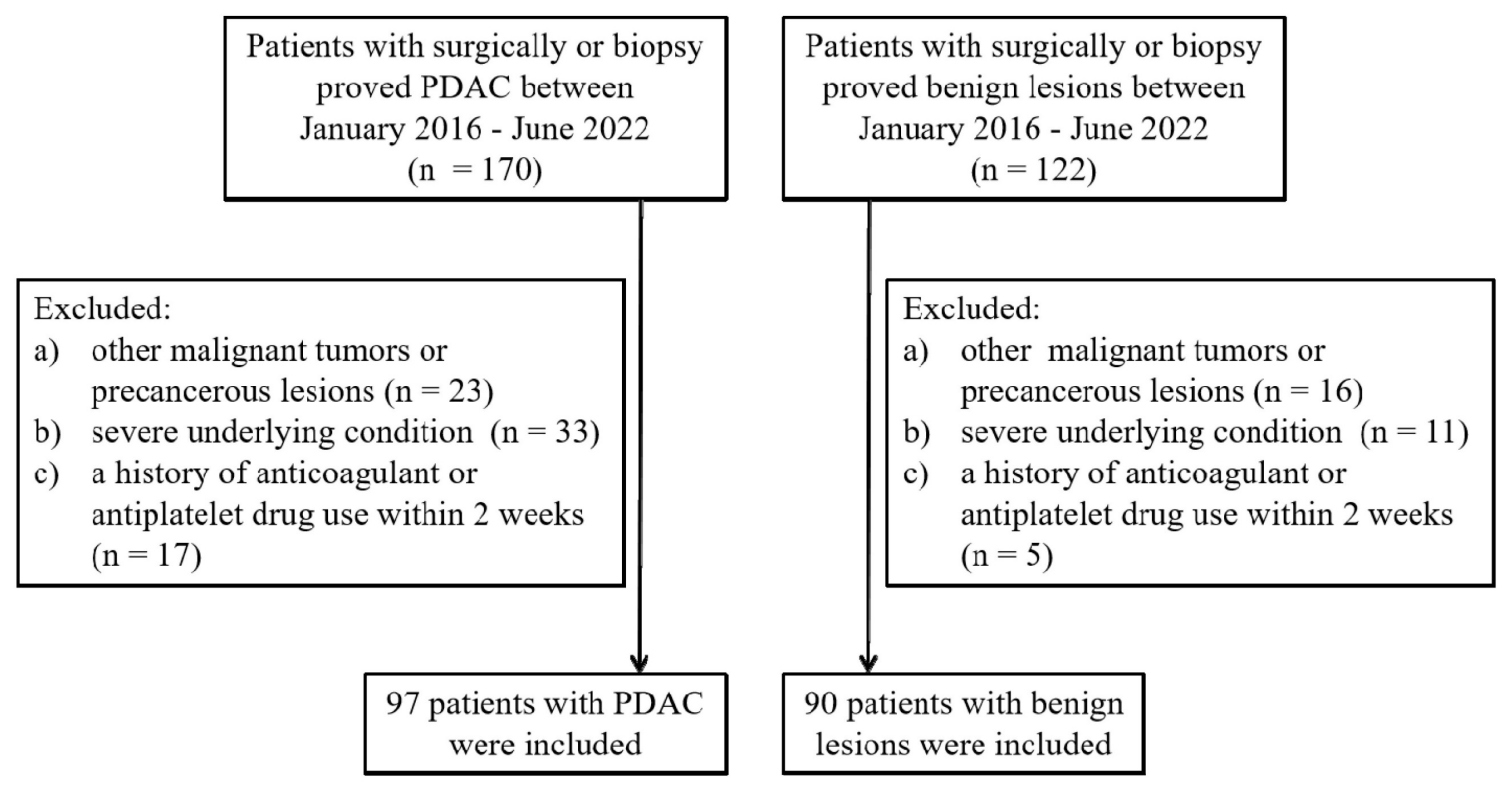
Flow diagram of patients' selection.

This study was approved by the institutional review board of the Affiliated Hospital of Nanjing University of Chinese Medicine, and patient informed consent was waived due to its retrospective design. The study comprised 97 patients with PDAC who were diagnosed through surgery or biopsy at the Affiliated Hospital of Nanjing University of Chinese Medicine from January 2016 to June 2022 (PDAC group). This group consisted of 48 males and 49 females, with a median age of 65.6 years (range: 47-83). Inclusion criteria were as follows: (1) patients with complete clinicopathological data, (2) patients with complete CT imaging data within 30 days before treatment, and (3) patients without distant metastasis. The exclusion criteria were: (1) patients with concurrent or prior malignant tumors, (2) patients with severe cardiovascular and cerebrovascular diseases, severe liver and kidney dysfunction, severe hematological and rheumatic immune system diseases, (3) a history of anticoagulant or antiplatelet drug use within 2 weeks before admission (**Figure 1**).

A total of 90 patients diagnosed with benign pancreatic diseases through surgery or biopsy (control group) were selected in the same period, consisting of 46 males and 44 females, with a median age of 63.0 years (range: 45-88). Among them, there were 17 chronic mass-forming pancreatitis, 23 pancreatic neuroendocrine tumors, 26 pancreatic cystadenomas, 19 intraductal papillary mucinous neoplasms, and 5 solid pseudopapillary tumors. Inclusion criteria were as follows: complete blood routine, coagulation function tests, and CT imaging examinations. Exclusion criteria were identical to those for PDAC (**Figure 1**).

### Clinical variables

Basic information and clinical variables of the subjects in the two groups were collected, including age, sex, hypertension, diabetes, smoking, drinking, platelet count (PLT), activated partial thromboplastin time (APTT), prothrombin time (PT), thrombin time (TT), fibrinogen (FIB), serum D-dimer, and CA19-9.

All patients were hospitalized, and blood samples were obtained from both PDAC and benign pancreatic lesion patients during their initial visit. Five milliliters of peripheral blood were drawn into a citrate test tube for the measurement of APTT, PT, TT, and FIB. The samples were stored at -20℃ for up to 1 day and measured using an automatic coagulation analyzer. Serum D-dimer levels were determined by a latex-enhanced immunoturbidimetric assay using a Sysmex CA 7000 (Sysmex Corp, Kobe, Japan) analyzer. CA19-9 levels were measured using an automated electrochemical luminescence immunoassay. Levels exceeding 37 U/mL were considered elevated for CA19-9.

### CT examination

All CT examinations were conducted using the Philips Brilliance 64 (Philips Healthcare, DA Best, the Netherlands) and Discovery HD750 (GE Healthcare, Milwaukee, Wisconsin, USA) following a standardized protocol. The CT scan parameters were as follows: tube voltage, 120 kVp; current, 200-400 mAs; pitch, 1.375; rotation speed, 0.75 s; slice thickness, 3.0 mm; slice interval, 3.0 mm; and a reconstruction interval of 1.25 mm. An initial cross-sectional nonenhanced CT scan was obtained, followed by a dynamic contrast-enhanced CT scan. The non-ionic contrast media Ultravist 300 (Bayer Schering Pharma AG, Berlin, Germany) was administrated intravenously (1.2-1.5 ml/kg) at a rate of 3.0 ml/s followed by 40 ml saline solution, using a power injector (Ulrich Medical, Ulm, Germany). After contrast agent injection, contrast-enhanced CT was performed in the arterial (35-40 seconds), portal venous (60-70 seconds), and delayed (110-130 seconds) phases. The scanning range extended from the level of the diaphragm to the pelvis.

### CT image analysis

Two board-certified abdominal radiologists (Y.L. and Y.C., with more than 5 years of experience in abdominal radiology) blinded to the clinicopathologic data independently reviewed the anonymized CT images. In case of any disagreement, a consensus was reached through discussion or referral to a third radiologist (Y.Z., with 10 years of abdominal radiology experience). The following CT imaging features were evaluated: tumor location, tumor size, pancreatic duct dilatation, common bile duct dilatation, calcification, tumor margin (well-defined or ill-defined margin), vascular invasion, lymph node enlargement, pancreatic atrophy, cystic components, and enhancement degree.

The tumor location was classified as head, neck, body, or tail. Pancreatic duct dilatation was defined as a maximal diameter of the main duct greater than 3 mm or a dilatation exceeding 2 mm larger than the narrowest visible portion. Common bile duct dilatation was confirmed when the maximum diameter of the common bile duct was 8 mm or larger. The longest tumor diameter was measured on axial images. Calcification was identified on unenhanced phase images, with CT values exceeding 100 Hounsfield units. A well-defined margin was characterized by a smooth and clearly visible boundary, while an ill-defined margin was indicated by spiculation or infiltration along more than 90% of the tumor perimeter 18. The criteria for vascular invasion included the presence of tumor thrombus, vessel occlusion, stenosis, or contour deformity, with more than half of the vessel perimeter in contact with the tumor 19. Lymph node enlargement was defined as a short-axis diameter greater than 10 mm 20. Parenchymal atrophy was considered present when the width of the upstream parenchyma was smaller than that of the normal pancreas 21. Cystic components within the tumor were identified if unenhanced tissue comprised more than 50% of the tumor volume 19. The degree of enhancement was classified into four levels: No enhancement was defined as an increase in CT value of less than 10 Hounsfield units; mild enhancement was defined as an increase between 10 and 30 Hounsfield units; moderate enhancement was defined as an increase between 30 and 50 Hounsfield units; and obvious enhancement was defined as an increase greater than 50 Hounsfield units.

### Statistical analysis

The data were analyzed using SPSS 26.0 and MedCalc 20.0.27. Descriptive statistics were computed for all variables. Continuous data were presented as the mean ± standard deviation. Categorical variables were presented as the number of cases (percentage). The Shapiro-Wilk test was used to test normally distributed continuous variables. Student's *t*-test or *U* test was applied to analyze continuous variables, depending on the normality of data distribution, while the χ2 or Fisher's exact test was used for categorical variables. The receiver operating characteristic (ROC) curve was generated to determine the area under the curve (AUC), sensitivity, specificity, accuracy, positive predictive value (PPV), and negative predictive value (NPV). The optimal cut-off value was calculated using the Youden index (sensitivity+specificity-1). Logistic regression analysis was performed to evaluate the performance of the features in differentiating benign pancreatic lesions from PDAC and to establish a prediction model based on the results of multivariate logistic regression analysis. The DeLong test was used to assess the statistical difference between the AUCs of the two models. A two-sided *P* value <0.05 was considered statistically significant for all tests.

## Results

### Patient characteristics

The clinical characteristics of the PDAC and control cohorts are summarized in **Table 1**. A total of 97 patients with pathologically confirmed PDAC and 90 patients with pathologically confirmed benign pancreatic lesions were included and compared in this study. The results are as follows: no significant differences were found between the two groups in terms of age, sex, PLT, APTT, PT, TT, hypertension, diabetes, smoking, or drinking. The levels of D-dimer (2.96 vs. 0.94 mg/L) and FIB (4.26 vs. 3.66 g/L) were significantly higher in the PDAC group than in the control group. Additionally, the PDAC group tended to have elevated CA19-9 levels compared with the control group (70.1% vs. 17.8%). Multivariate logistic regression analysis of the clinical variables showed that D-dimer and CA19-9 are independent predictors for differentiating PDAC from benign pancreatic lesions (*P* = 0.002, *P* < 0.001, respectively) (**Table S1**).

### Comparison of imaging features between PDAC and benign pancreatic lesions

Imaging findings between PDAC and benign pancreatic lesions are summarized in **Table 2**. Descriptive statistics showed that cystic components (63.3% vs. 24.7%), a well-defined margin (67.8% vs. 33.0%), and moderate to significant enhancement (30.0% vs. 14.4%) were more common in benign pancreatic lesions than in PDAC. Pancreatic duct dilatation (63.9% vs. 35.6%), common bile duct dilatation (36.1% vs. 14.4%), vascular invasion (43.3% vs. 27.8%), lymph node enlargement (33.0% vs. 6.7%), and pancreatic atrophy (34.0% vs. 7.8%) were significantly more frequent in PDAC than in benign pancreatic lesions. No significant differences were found in tumor location, tumor size, or calcification between the two groups. Multivariate logistic regression analysis of the imaging variables showed that tumor margin, lymph node enlargement, pancreatic atrophy, cystic components, and enhancement degree are potential independent predictors for differentiating PDAC from benign pancreatic lesions (*P* = 0.006, *P* = 0.001, *P* = 0.001, *P* = 0.002, *P* = 0.047, respectively) (**Table S2**).

### Clinical model

Among the 13 clinical features assessed, two independent predictors, CA19-9 and serum D-dimer, were identified. The AUC values for CA19-9 and serum D-dimer were 0.76 (95% CI: 0.69-0.83) and 0.76 (95% CI: 0.69-0.83), respectively. The sensitivities were 70.1% and 72.2%, while the specificities were 82.2% and 71.1%, respectively. The accuracies were 75.9% and 71.7%, with positive predictive values (PPVs) of 80.9% and 72.9%, and negative predictive values (NPVs) of 71.8% and 70.4%. The optimal cut-off value for serum D-dimer to differentiate benign pancreatic lesions from PDAC was 0.84 mg/L, according to the Youden index. The combined model, referred to as Model 1, incorporating these two indicators, yielded an AUC value of 0.86 (95% CI: 0.81-0.91), with 76.3% sensitivity, 82.2% specificity, 79.1% accuracy, 82.2% PPV, and 76.3% NPV, respectively.

### Radiologic model

Among the imaging variables assessed, five independent predictors for the risk of PDAC were identified: tumor margin, lymph node enlargement, pancreatic atrophy, cystic components, and enhancement degree. The AUC values for these predictors were 0.67 (95% CI: 0.60-0.75), 0.63 (95% CI: 0.55-0.71), 0.63 (95% CI: 0.55-0.71), 0.69 (95% CI: 0.62-0.77), and 0.58 (95% CI: 0.50-0.66), respectively. Sensitivities ranged from 33.0% to 85.6%, while specificities ranged from 30.0% to 93.3%. The accuracies varied from 58.8% to 69.5%, with PPVs ranging from 56.9% to 84.1% and NPVs ranging from 56.4% to 70.4%. When these five indicators were combined to differentiate PDAC from benign pancreatic lesions, the AUC reached a maximum value of 0.85 (95% CI: 0.79-0.90). The sensitivity, specificity, accuracy, PPV, and NPV of the combined model, referred to as Model 2, were 70.1%, 85.6%, 77.6%, 84.0%, and 72.6%, respectively. These values were all higher than those of each single indicator.

### Overall model (combined clinical and radiologic models)

**Figure 2:**
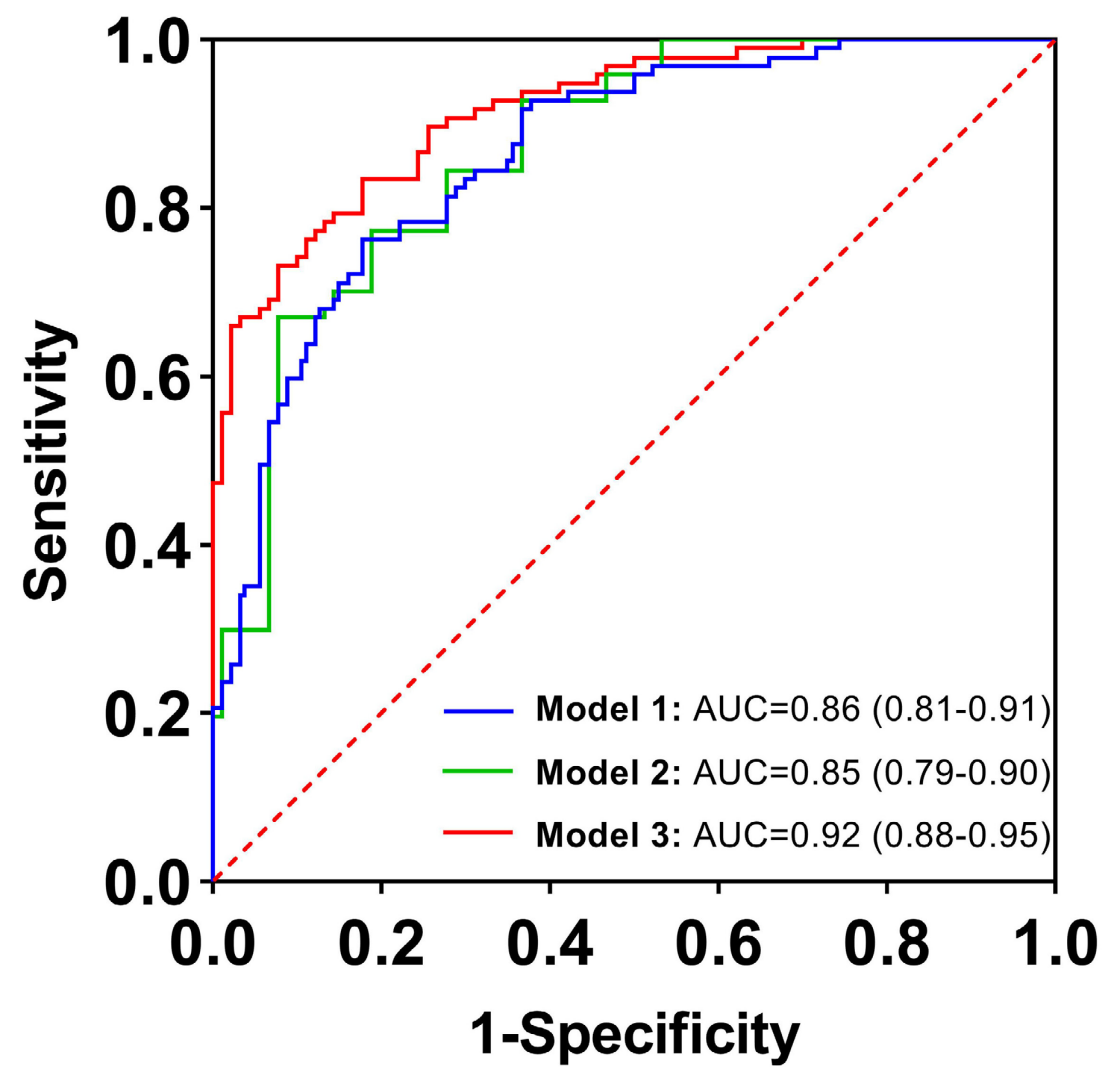
Receiver operating characteristic curves for diagnostic performance in distinguishing between benign pancreatic lesions and pancreatic cancer with clinical (Model 1), imaging (Model 2), and combination of clinical and imaging features (Model 3). The areas under the curve were 0.86, 0.85, and 0.92, respectively.

**Figure 3:**
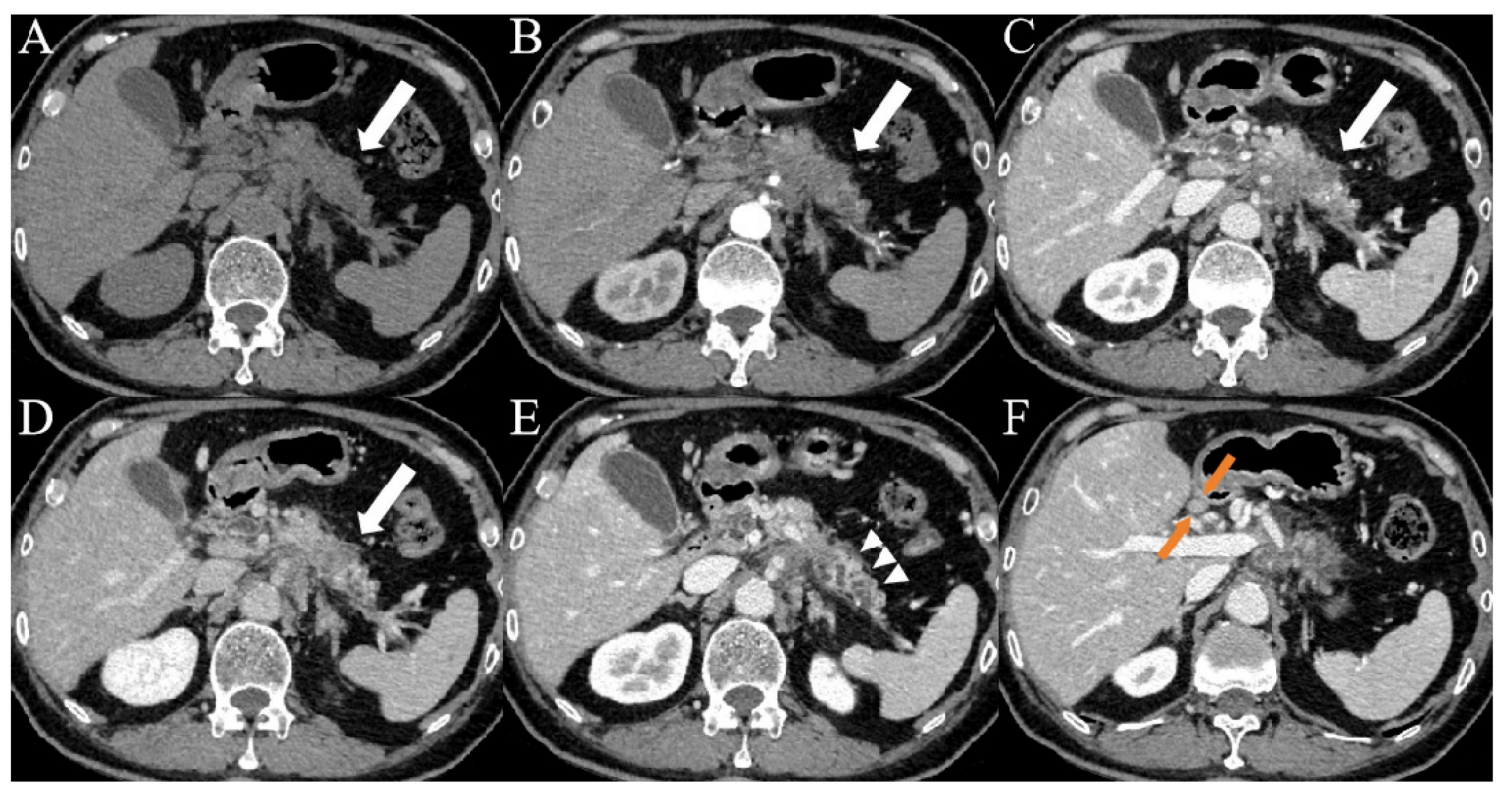
Images from a patient with PDAC. (A-D) CT images showed patchy areas of low density within the body of the pancreas (white arrows) with a hypovascular pattern, the margin of which was ill-defined. (E) Contrast-enhanced CT images during the portal venous phase showed duct dilatation and marked tail atrophy (arrowheads). (F) CT imaging showed a significantly enlarged lymph node (orange arrows) in the hepatogastric space, with a short axis measuring approximately 1.1 cm. The patient's CA19-9 level was 78.3 U/mL (> 37 U/mL), and the D-dimer level was 14.7 mg/L (> 0.84 mg/L).

**Figure 4:**
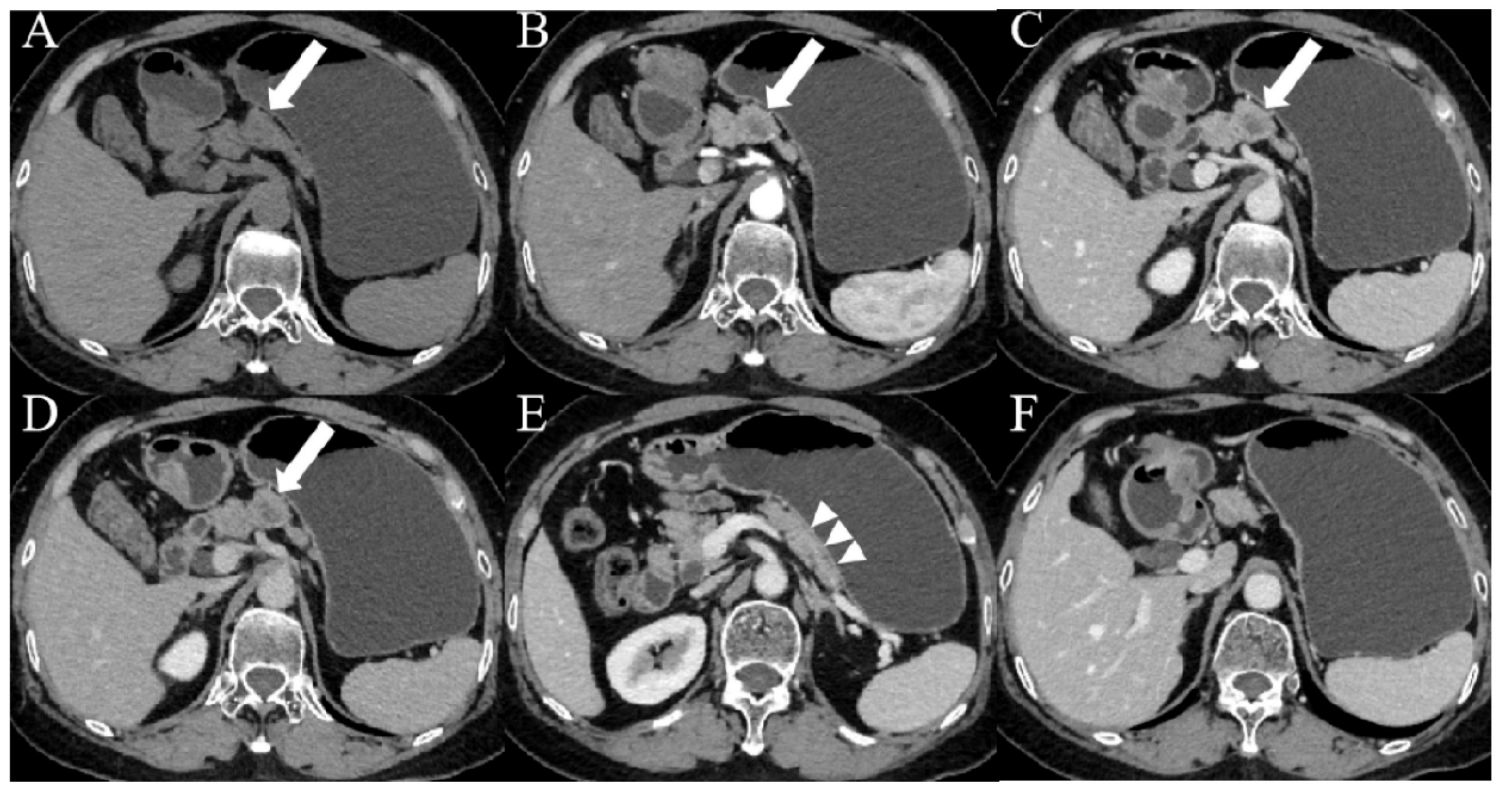
Images from a patient with pNET G2. (A-D) CT images showed a nodular, slightly enhanced area (white arrows) in the body of the pancreas, with internal cystic non-enhancing regions and an indistinct margin. (E) CT imaging showed a normal pancreatic tail without atrophy (arrowheads). (F) The fat spaces around the pancreas were clear, with no markedly enlarged lymph nodes. The patient's CA19-9 level was 19.6 U/mL (< 37 U/mL), and the D-dimer level was 0.23 mg/L (< 0.84 mg/L).

When clinical and imaging indicators were combined, five indicators were identified as independent predictors based on the results of multivariable logistic regression in **Table 3**: CA19-9, serum D-dimer, lymph node enlargement, pancreatic atrophy, and cystic components. The combined model, incorporating these five indicators, was named Model 3. The AUC value was 0.92 (95% CI: 0.88-0.95), the sensitivity was 83.5%, the specificity was 82.2%, the accuracy was 82.9%, the PPV was 83.5%, and the NPV was 82.2%. There was a significant difference in AUCs between Model 3 and the other two models according to the DeLong test (*P* = 0.0067, *P* = 0.0038, respectively) (**Table 4**;** Figure 2**). The clinical applications of Model 3 were illustrated in **Figure 3** and **Figure 4**, with the predicted results of the model aligning with the pathological findings.

## Discussion

Early detection of surgically resectable neoplasms remains the most effective strategy for improving PDAC survival rates and achieving potential cure. Most long-term survivors are typically diagnosed at Stage I through incidental imaging findings, often presenting with smaller tumors that are associated with better survival outcomes 3, 22. However, the relatively low prevalence of pancreatic cancer compared to other lethal malignancies has limited the widespread implementation of pancreas surveillance programs 23. Therefore, there is an urgent need for more efficient, accurate, and less invasive diagnostic methods to expedite treatment initiation and minimize unnecessary interventions 24.

Our study demonstrated that CA19-9, serum D-dimer, lymph node enlargement, pancreatic atrophy, and cystic components are statistically significant independent variables for distinguishing PDAC from benign pancreatic lesions. The clinical-radiologic model incorporating these five indicators exhibits superior diagnostic performance compared to standalone clinical or imaging models, as reflected by the AUC values. Additionally, we found that serum D-dimer holds significant clinical value in the differential diagnosis of PDAC, with an optimal diagnostic cut-off of 0.84 mg/L, a novel finding not reported in previous studies. In addition, the combined use of D-dimer and CA19-9 can enhance the diagnostic value of CA19-9.

CA19-9 is widely used as a serum biomarker for pancreatic cancer detection, but its utility is limited by several factors. Its sensitivity is low, as elevated levels are typically observed in the later stages of the disease. Furthermore, its specificity is suboptimal, as CA19-9 levels can also rise in non-pancreatic cancers and benign conditions. Additionally, approximately 10% of the population is genetically negative for CA19-9 25. However, recent studies have demonstrated that setting cutoff levels of CA19-9 based on single nucleotide polymorphisms (SNPs) improves CA19-9's diagnostic sensitivity for resectable-stage pancreatic cancer, increasing it from 53% to 61% at 99% specificity 26. To further enhance the diagnostic accuracy of CA19-9, its combined use with imaging factors has been investigated in several studies 27.

Durczynski et al compared plasma D-dimer levels in portal and peripheral blood from pancreatic cancer patients without venous thromboembolism, and found that the mean D-dimer values were higher in portal than in peripheral blood. Their findings showed a close association between the activation of hemostasis, reflected by elevated D-dimers in portal blood, and the presence of pancreatic cancer. Moreover, measuring D-dimer levels in portal blood may represent a promising approach for pancreatic cancer screening 28. Li et al conducted a comprehensive analysis of conventional coagulation and thromboelastography indicators in benign and malignant pancreatic diseases. They found that FIB, D-dimer, maximum amplitude, Angle, and coagulation index were effective in the early diagnosis of PDAC. Furthermore, they discovered that combining CA19-9 with coagulation indicators could significantly improve the early diagnostic performance of CA19-9 29.

Pancreatic cancer is highly malignant and prone to early metastasis, with the lymphatic system serving as a major route for metastasis. Lymph node metastases are observed in 50-70% of pancreatic cancer patients, likely due to the dense lymphatic networks surrounding the pancreas 30. This is commonly manifested as enlarged lymph nodes around the pancreas on imaging. In addition, the pancreas distal to the tumor often shows signs of atrophy. PDAC triggers a process in which the acinar cell layer thins due to disrupted flow within the pancreatic duct. This thinning results in cell death, either by apoptosis or necrosis. The loss of acinar cells then leads to fibrotic changes and immune cell infiltration. These alterations collectively manifest as pancreatic parenchymal atrophy 31. Miura et al examined 20 patients who had undergone pancreatectomy for a single localized stricture of the main pancreatic duct, with no identifiable masses on imaging. The cohort was divided into two groups: 10 patients diagnosed with pancreatic ductal adenocarcinoma (cancer group) and 10 patients with benign strictures (benign group). Computed tomography revealed that focal parenchymal atrophy and fat replacement were more frequently observed in the cancer group (7/10) compared to the benign group (1/10) (*P* = 0.02). These findings suggest that focal parenchymal atrophy and fat replacement may serve as indicators for the early detection of pancreatic cancer 32. Yamao K et al assessed the CT findings associated with small pancreatic cancer (≤10 mm in diameter), including carcinoma in situ (CIS), and compared them to benign main pancreatic duct (MPD) stenosis. They found that partial pancreatic parenchymal atrophy (PPA), upstream PPA, and MPD abrupt stenosis on CT images were highly suggestive of small pancreatic cancers, including CIS 33.

PDAC is one of the most aggressive solid tumors in humans. Histologically, it is characterized by a dense stromal matrix composed of various cellular and acellular components. This stromal matrix, often referred to as a desmoplastic reaction or tumor microenvironment, is relatively dense and lacks a cystic structure, making cystic changes less likely 34. Therefore, on CT scans, PDAC typically appears as a mildly enhancing mass with soft tissue density.

PDAC is frequently located in the head of the pancreas, where mass compression often results in ductal obstruction and secondary dilation of both the common bile duct and pancreatic duct 5, 6. In our study, bile duct and pancreatic duct dilation were significantly more common in pancreatic cancer compared to benign pancreatic lesions (36.1% vs. 14.4%, 63.9% vs. 35.6%, respectively). Given the strong potential correlation between the bile duct and pancreatic duct dilation, both variables were excluded from the multifactorial regression analysis.

There are some limitations to our study. Due to its retrospective nature, selection bias was inevitable. Moreover, in comparing benign and malignant pancreatic lesions, we included only pancreatic ductal adenocarcinoma in the malignant group, as other pathological types of pancreatic cancer are relatively rare. In future studies, we plan to expand the sample size and incorporate other types of pancreatic cancer for more comprehensive analyses. Finally, we did not include CT data from normal tissues. Incorporating normal tissues as a comparison group would enhance the comprehensive analysis of the imaging characteristics of pancreatic cancer for the differences between normal tissues and benign lesions. Therefore, our future studies will consider including normal tissues as a comparison group to further investigate the imaging differences among pancreatic cancer, normal tissues, and benign pancreatic lesions. Finally, a multicenter program involving more patients is needed, and external validation is required to confirm the robustness and generalizability of our model.

In conclusion, our study demonstrates that a combined clinical-radiologic model incorporating serum D-dimer, CA19-9, lymph node enlargement, pancreatic atrophy, and cystic components shows high sensitivity and specificity in differentiating between PDAC and benign pancreatic lesions. This predictive model holds potential as a valuable decision support tool for clinicians and radiologists, aiding in the early detection of pancreatic cancer and potentially improving patient prognosis.

## Supplementary Material

Supplementary tables.

## Figures and Tables

**Table 1:** Clinical characteristics

Variables	PDAC group (n=97)	Control group (n=90)	*p* value
Age (years)*	65.6 ± 9.77	63.0 ± 10.2	0.083
Sex			0.824
Male	48 (49.5)	46 (51.1)	
Female	49 (50.5)	44 (48.9)	
Serum D-dimer (mg/L)*	2.96 ± 3.45	0.94 ± 0.98	< 0.001
FIB (g/L)	4.26 ± 0.91	3.66 ± 1.06	< 0.001
CA19-9 level (U/mL)*			< 0.001
≥37	68 (70.1)	16 (17.8)	
<37	29 (29.9)	74 (82.2)	
PLT (10^9/L)*	186.7 ± 61.2	184.8 ± 62.7	0.839
APTT (s)*	37.1 ± 4.99	37.8 ± 4.51	0.336
PT (s)*	13.7 ± 1.33	13.7 ± 2.02	0.827
TT (s)*	17.0 ± 1.47	17.2 ± 1.34	0.280
Hypertension			0.772
Yes	31 (32.0)	27 (30.0)	
No	66 (68.0)	63 (70.0)	
Diabetes			0.267
Yes	11 (11.3)	6 (6.7)	
No	86 (88.7)	84 (93.3)	
Smoking			0.545
Yes	16 (16.5)	12 (13.3)	
No	81 (83.5)	78 (86.7)	
Drinking			0.890
Yes	25 (25.8)	24 (26.7)	
No	72 (74.2)	66 (73.3)	

Note: Data are numbers of lesions with percentages. *FIB* fibrinogen, *CA19-9* carbohydrate antigen 19-9, *PLT* platelet, *APTT* activated partial thromboplastin time, *PT* prothrombin time*, TT* thrombin time *Data are mean ± standard deviation

**Table 2:** Imaging variables

Variables	PDAC group (n=97)	Control group (n=90)	*p* value
Location			0.934
Head/neck	62 (63.9)	57 (63.3)	
Body/tail	35 (36.1)	33 (36.7)	
Diameter (mm)*	35.5 ± 17.5	31.1 ± 16.8	0.079
Calcification			0.052
Yes	5 (5.2)	12 (13.3)	
No	92 (94.8)	78 (86.7)	
PD dilatation			< 0.001
Yes	62 (63.9)	32 (35.6)	
No	35 (36.1)	58 (64.4)	
CBD dilatation			0.001
Yes	62 (63.9)	13 (14.4)	
No	35 (36.1)	77 (85.6)	
Tumor margin			< 0.001
well-defined	32 (33.0)	61 (67.8)	
ill-defined	65 (67.0)	29 (32.2)	
Vascular invasion			0.027
Yes	42 (43.3)	25 (27.8)	
No	55 (56.7)	65 (72.2)	
Lymph node enlargement			< 0.001
Yes	65 (67.0)	6 (6.7)	
No	32 (33.0)	84 (93.3)	
Pancreatic atrophy			< 0.001
Yes	33 (34.0)	7 (7.8)	
No	64 (66.0)	83 (92.2)	
Cystic components			< 0.001
Yes	24 (24.7)	57 (63.3)	
No	73 (75.3)	33 (36.7)	
Enhancement degree			0.010
No/Mild	83 (85.6)	63 (70.0)	
Moderate/Significant	14 (14.4)	27 (30.0)	

Note: Data are numbers of lesions with percentages. *PD* pancreatic duct, *CBD* common bile duct *Data are mean ± standard deviation

**Table 3:** Univariable and multivariable logistic regression analysis of potential predictors

	Univariable		Multivariable	
	OR (95% CI)	*p* value	OR (95% CI)	*p* value
Clinical variables				
Serum D-dimer (mg/L)	1.79 (1.36, 2.36)	<0.001	1.67 (1.18, 2.36)	0.004
FIB (g/L)	1.93 (1.37, 2.72)	<0.001	1.38 (0.86, 2.21)	0.184
CA19-9 level (U/mL)	10.9 (5.40, 21.7)	<0.001	9.38 (3.58, 24.6)	<0.001
Imaging variables				
PD dilatation	3.21 (1.77, 5.84)	<0.001	2.67 (0.97, 7.37)	0.057
CBD dilatation	3.34 (1.63, 6.86)	0.001	2.74 (0.91, 8.29)	0.074
Tumor margin	4.27 (2.32, 7.88)	<0.001	1.66 (0.65, 4.24)	0.292
Vascular invasion	1.99 (1.08, 3.66)	0.028	0.64 (0.23, 1.81)	0.402
Lymph node enlargement	6.89 (2.72, 17.5)	<0.001	3.82 (1.06, 13.8)	0.041
Pancreatic atrophy	6.11 (2.54, 14.7)	<0.001	7.06 (1.93, 25.8)	0.003
Cystic components	0.19 (0.10, 0.36)	<0.001	0.23 (0.08, 0.63)	0.004
Enhancement degree	0.39 (0.19, 0.81)	0.012	0.34 (0.10, 1.20)	0.095

Note: *FIB* fibrinogen, *CA19-9* carbohydrate antigen 19-9, *PD* pancreatic duct, *CBD* common bile duct, *OR* odds ratio, *CI* confidence interval

**Table 4:** Diagnostic performance of prediction models

	AUC*	Sensitivity	Specificity	Accuracy	PPV	NPV
Model 1	0.86 (0.81, 0.91)	76.3	82.2	79.1	82.2	76.3
Model 2	0.84 (0.79, 0.90)	70.1	85.6	77.6	84.0	72.6
Model 3	0.92 (0.88, 0.95)	83.5	82.2	82.9	83.5	82.2

Note: Except where indicated, data are percentages, with numbers of patients in parentheses. Performance is presented as AUC, sensitivity, and specificity values according to the optimal selected cut-off. AUC = area under the receiver operating characteristic curve, PPV = positive predictive value, NPV = negative predictive value. * Data are AUCs, with 95% CIs in parentheses.

## Abbreviations

CT: computed tomography
PDAC: pancreatic ductal adenocarcinoma
CA19-9: carbohydrate antigen 19-9
ROC: receiver operating characteristic
AUC: area under the curve
pNET: pancreatic neuroendocrine tumor
PLT: platelet
APTT: activated partial thromboplastin time
PT: prothrombin time
TT: thrombin time
FIB: fibrinogen
PD: pancreatic duct
CBD: common bile duct
PPV: positive predictive value
NPV: negative predictive value
OR: odds ratio
CI: confidence interval
