# Supplementary material for: Diagnostic Value of Serum D-dimer, CA19-9, and CT Imaging Features in Pancreatic Ductal Adenocarcinoma and Benign Pancreatic Lesions
Source: J Cancer. 2025 Jun 12;16(9):2812–21. doi: 10.7150/jca.111548 (PMC12244095; doi:10.7150/jca.111548)
Supplement: Supplementary file 1 — Supplementary tables. [file jcav16p2812s1.pdf]

Supplementary information

Table S1 Univariable and multivariable logistic regression analysis of clinical variables

Clinical variables	Univariable		Multivariable	
	OR (95% CI)	*p* value	OR (95% CI)	*p* value
Serum D-dimer (mg/L)	1.79 (1.36, 2.36)	<0.001	1.54 (1.16, 2.04)	0.002
FIB (g/L)	1.93 (1.37, 2.72)	<0.001	1.34 (0.90, 1.99)	0.149
CA19-9 level (U/mL)	10.9 (5.42, 21.7)	<0.001	8.89 (4.24, 18.7)	<0.001

Note: *FIB* fibrinogen, *CA19-9* carbohydrate antigen 19-9, *OR* odds ratio, *CI* confidence interval

Table S2 Univariable and multivariable logistic regression analysis of imaging variables

Imaging variables	Univariable		Multivariable	
	OR (95% CI)	*p* value	OR (95% CI)	*p* value
PD dilatation	3.21 (1.77, 5.84)	<0.001	1.77 (0.78, 3.99)	0.171
CBD dilatation	3.34 (1.63, 6.86)	0.001	2.20 (0.87, 5.57)	0.097
Tumor margin	4.27 (2.32, 7.88)	<0.001	2.85 (1.35, 6.02)	0.006
Vascular invasion	1.99 (1.08, 3.66)	0.028	0.78 (0.33, 1.85)	0.576
Lymph node enlargement	6.89 (2.72, 17.5)	<0.001	5.95 (2.00, 17.7)	0.001
Pancreatic atrophy	6.11 (2.54, 14.7)	<0.001	5.55 (1.93, 16.0)	0.001
Cystic components	0.19 (0.10,0.36)	<0.001	0.29 (0.13, 0.64)	0.002
Enhancement degree	0.39 (0.19, 0.81)	0.012	0.40 (0.16, 0.99)	0.047

Note: *PD* pancreatic duct, *CBD* common bile duct, *OR* odds ratio, *CI* confidence interval
